# RETRACTION: Effect of Adjunctive Prophylactic Macrolides Used at the Caesarean Section on Endometritis and Surgical Site Wound Infection: A Meta‐Analysis

**DOI:** 10.1111/iwj.70221

**Published:** 2025-02-11

## Abstract

**RETRACTION**: ZhangT. , WangJ. , HuaZ. , YaoX. , ZhangF. , and ZhouY. , “Effect of Adjunctive Prophylactic Macrolides Used at the Caesarean Section on Endometritis and Surgical Site Wound Infection: A Meta‐Analysis,” International Wound Journal 20, no. 8 (2023): 3307–3314, 10.1111/iwj.14211.37161646 PMC10502253

The above article, published online on 09 May 2023, in Wiley Online Library (http://onlinelibrary.wiley.com/), has been retracted by agreement between the journal Editor in Chief, Professor Keith Harding; and John Wiley & Sons Ltd. Following an investigation by the publisher, all parties have concluded that this article was accepted solely on the basis of a compromised peer review process. In addition, the investigation found unattributed textual overlap within the introduction and discussion sections between this article and another article by different authors [1]. The editors have therefore decided to retract the article. The authors responded to our notice regarding the retraction and stated that textual overlap was unintentional and that the core data, methodology, and conclusions of the manuscript are original.

**Reference:**

[1] FarmerN. , Hodgetts‐MortonV. , MorrisR. K. , “Are Prophylactic Adjunctive Macrolides Efficacious Against Caesarean Section Surgical Site Infection: A Systematic Review and Meta‐Analysis,” European Journal of Obstetrics & Gynecology and Reproductive Biology 244 (2020): 163–171, 10.1016/j.ejogrb.2019.11.026.31810022

**RETRACTION**: T. Zhang , J. Wang , Z. Hua , X. Yao , F. Zhang , and Y. Zhou , “Effect of Adjunctive Prophylactic Macrolides Used at the Caesarean Section on Endometritis and Surgical Site Wound Infection: A Meta‐Analysis,” International Wound Journal 20, no. 8 (2023): 3307–3314, 10.1111/iwj.14211.37161646 PMC10502253

The above article, published online on 09 May 2023, in Wiley Online Library (http://onlinelibrary.wiley.com/), has been retracted by agreement between the journal Editor in Chief, Professor Keith Harding; and John Wiley & Sons Ltd. Following an investigation by the publisher, all parties have concluded that this article was accepted solely on the basis of a compromised peer review process. In addition, the investigation found unattributed textual overlap within the introduction and discussion sections between this article and another article by different authors [1]. The editors have therefore decided to retract the article. The authors responded to our notice regarding the retraction and stated that textual overlap was unintentional and that the core data, methodology, and conclusions of the manuscript are original.

## Reference

[1] N. Farmer , V. Hodgetts‐Morton , R. K. Morris , “Are Prophylactic Adjunctive Macrolides Efficacious Against Caesarean Section Surgical Site Infection: A Systematic Review and Meta‐Analysis,” European Journal of Obstetrics & Gynecology and Reproductive Biology 244 (2020): 163–171, 10.1016/j.ejogrb.2019.11.026.31810022

